# Atrial Fibrillation-Induced Cardiac Shock: First Manifestation of a Congenitally Corrected Transposition of the Great Arteries in a 45-Year-Old Man

**DOI:** 10.1155/2012/126764

**Published:** 2012-11-24

**Authors:** M. Graf, M. Zaczkiewicz, J. Torzewski, O. Zimmermann

**Affiliations:** Cardiovascular Center Oberallgäu-Kempten, Sana Kliniken AG, Clinic Immenstadt, 87509 Immenstadt, Germany

## Abstract

*Background.* The congenitally corrected transposition of the great arteries (L-TGA) is a very rare congenital heart defect, which often remains undetected for several decades of life. *Case Presentation.* We report on a 45-year-old man without prior history of heart disease, presenting with cardiac shock related to a first episode of tachycardic atrial fibrillation. The diagnostic work-up identified a L-TGA as the underlying cause for acute heart failure. *Discussion.* L-TGA is a very rare congenital heart defect, which is characterized by an atrioventricular as well as a ventriculoarterial discordance. By this means, the physiological sequence of pulmonary and systemic circulation is still maintained. On the basis of an ongoing strain of the right ventricle, which has to carry the burden of the systemic blood pressure, after more than four decades without symptoms, acute heart failure was triggered by a tachycardic atrial fibrillation.

## 1. Introduction

The congenitally corrected transposition of the great arteries (L-TGA) is a very rare congenital heart defect that represents less than 1% of all congenital heart disorders [1]. In many cases, these patients remain asymptomatic for several decades of life. We report the case of a 45-year-old man, who was admitted to our emergency unit due to haemodynamic instable tachycardic atrial fibrillation. Further diagnostics revealed an—so far unknown—L-TGA.

## 2. Case Presentation

A 45-year-old man was admitted to our emergency unit because of rapidly increasing dyspnoea and pulmonary rales. The patient's history lacked previous significant illnesses, particularly structural heart or lung diseases and cardiovascular risk factors. The patient described himself up to now as sportive and physically trained, but remarked a general weakness within the last couple of weeks. Since 48 h he sensed palpitations which had now ended up in tachycardia.

**Figure 1:**
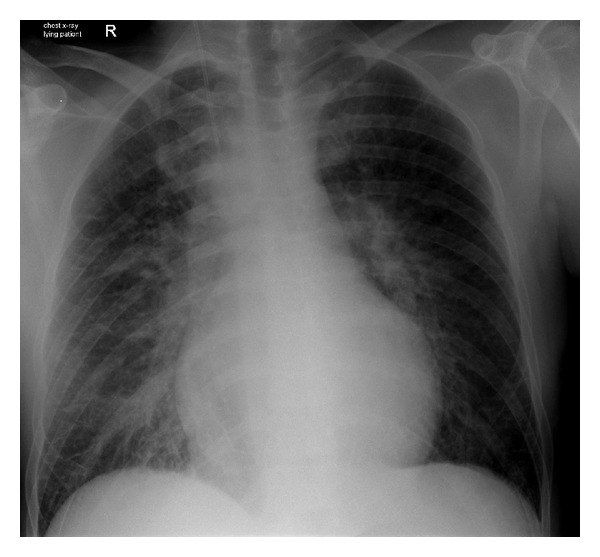
Chest X-ray of the lying patient in the a.p. path of ray. The congestion of the pulmonary veins reflects the cardiac shock and mild pulmonary edema. A meso-/dextrocardia with a dilated heart silhouette is the prominent finding.

The electrocardiogram (ECG) showed tachycardic atrial fibrillation with a mean heart rate of 160 bpm. The initial systolic blood pressure amounted not more than 80 mmHg. Under the clinical presentation of acute heart failure, we immediately performed an electrical cardioversion. After two attempts with biphasic application of 150 and 200 Joule of energy, a normofrequent sinus rhythm could be established. Intermitting short episodes of an AV-junctional rhythm were observed. Even now, the patient still showed signs of an ongoing cardiac shock and remained dependent on constant dobutamine supply. Although an ameliorated spontaneous breathing was reported, orthopnoea persisted under the treatment with 6 liter/minute oxygen flow. The chest X-ray demonstrated pulmonary vein stasis and a previously unknown meso-/dextrocardia with a dilated heart silhouette (Figure 1). Relevant structural pulmonary abnormalities were excluded.

**Figure 2:**
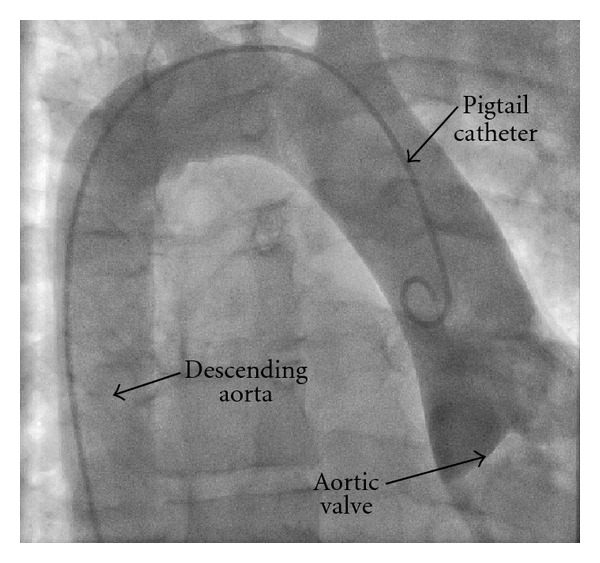
The aortic angiography performed during cardiac catheterisation confirms the dextrocardia and dextroposition of the aorta.

As signs of cardiac shock proceeded and slightly elevated levels of high sensitive troponin T (0.020 ng/mL; reference value < 0.015 ng/mL) could be noticed, an indication for a coronary angiography was seen. This diagnostic procedure was technically demanding because of a dextroposition of the aorta (Figure 2). Finally, a significant coronary heart disease could be excluded. We renounced the performance of an additional laevocardiography because of the technical difficulties, as well as the patient's persisting dyspnea and instable hemodynamics.

**Figure 3:**
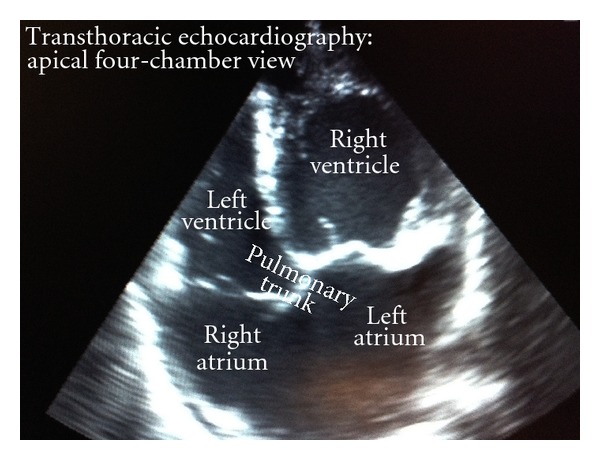
The transthoracical echocardiography (here apical four-chamber view) demonstrates the in situ anatomy in L-TGA. Both ventricles changed their position. The right ventricle shows dilation, hypertrophy, and trabecularisation. The right atrioventricular valve is located closer to the apex compared with the left atrioventricular valve.

The ECG showed now a first degree atrioventricular block and a right bundle branch block. Clinical chemistry pointed to an increased D-dimer of 1.3 *μ*g/mL (reference value < 0.5 *μ*g/mL). Given the insufficient systemic bloodpressure, a bedside echocardiography was performed in order to exclude signs of acute right heart failure under the suspicion of a potential pulmonary embolism (Figure 3). Thereby, we also aimed to exclude competing causes for acute heart failure, such as valve abnormalities or pericardial effusion. For the reason of dextrocardia, the echocardiographic examination had to be performed from the right chest side. The atrium and ventricle, preceding the pulmonary arterial bed, were not dilated. For a start, fulminant pulmonary embolism seemed to be unlikely and was not considered to explain the patient's condition.

On contrary, we found a massive dilatation (end-diastolic diameter 77 mm) and a highly reduced ejection fraction (approximately EF = 20%) of the left-sided ventricle, that is, the one in charge to support the systemic circulation. Due to the distinct dilatation of the left-sided ventricle, the atrioventricular valve showed a moderate insufficiency. Interestingly, the ventricle itself appeared remarkably trabeculated. Furthermore, the echocardiographical standard views demonstrated an abnormal origin of the right and left ventricular outflow tract. Taking this into account and being aware of the difficulties during coronary angiography, we wanted to exclude a confusion of both ventricles in this abnormal echocardiography setting. For this reason, we injected agitated saline over a central venous catheter into the right jugular vein. We were affirmed in our former findings, as the saline-induced *bubbles* were detectable by echocardiography in the slim, right-sided chambers only. As the *bubbles* were strictly located on the right side of the heart, without any signs of wash-out, there was no evidence for a shunt flow through the atrial or ventricular septum.

**Figure 4:**
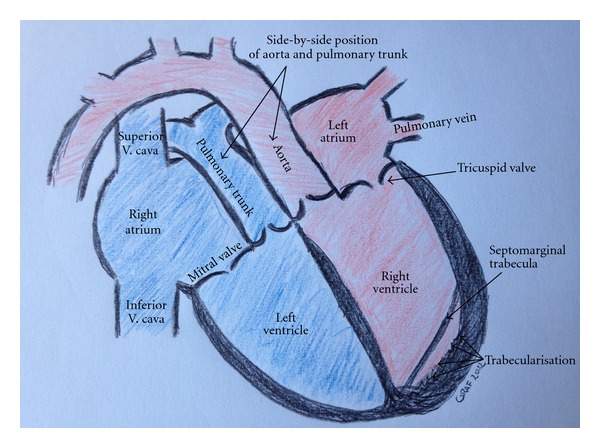
A schematic overview of the anatomical structures in this case of L-TGA is given.

In a final step, cardiac magnetic resonance imaging (MRI) could confirm the echocardiographic findings and identified the underlying structural heart disease as a congenitally corrected transposition of the great arteries (L-TGA; schematic overview: see Figure 4). In order to maintain a stable sinus rhythm, amiodarone was administered with a total loading dose of 5 g within the first five days, and the treatment was afterwards continued with a maintenance dose of 200 mg per day. Additionally, medicamentous heart failure therapy was begun, using a beta-blocker, spironolactone, and diuretics. Within the next couple of days, the catecholamine supply could be reduced stepwise and was finally stopped after 6 days of treatment. Having received stable cardiopulmonary conditions, the patient was referred to a heart transplantation program, where he was listed for organ transplantation.

## 3. Discussion

The congenitally corrected transposition of the great arteries (L-TGA) represents a very rare congenital heart defect [1]. It is characterized by an atrioventricular discordance on the one hand, and a ventriculoarterial discordance on the other hand. Besides a switch of the great arteries—as to be concluded from the name TGA—the physiological sequence of pulmonary and systemic circulation is still maintained due to a simultaneous inversion of both ventricles, that is, congenitally corrected. A schematic overview of these anatomical findings is depicted in Figure 4.

The systemic venous and deoxygenated blood is collected by the venae cavae in the right atrium. The right atrium is linked with a morphologically left-sided ventricle that is now positioned on the right heart side. As to be explained by embryological means, in case of inversion of the ventricles, the atrioventricular valve always goes with the ventricle, so that the valve between right atrium and left ventricle is now the mitral valve. The hypotrophied left ventricle pumps the blood through the discordantly linked pulmonary trunk into the pulmonary arteries. The left atrium gets the oxygenated blood from the lungs and leads it through the tricuspid valve into the left-sided, but morphologically right ventricle. Due to the increased contractility which is necessary to maintain the systemic circulation, the originally right-sided ventricle becomes dilated. However, a couple of echocardiographical signs still indicate its right-sided origin, for example, the distinct trabecularisation, the existence of a septomarginal trabecular, and a more apical insertion of the atrioventricular valve on the left side compared with the right heart side [2]. From the originally right ventricle, the blood runs through the aortic valve into the ascending aorta.

Further congenital anomalies, for example, septum defects or valve defects, can occur facultatively [3–5] as shown in Table 1. In contrast to the *common* (D-)TGA, a ventricular septum defect is not necessary for the maintenance of a sufficient circulation and blood oxygenation. In our patient we could even exclude a septal defect-related shunt flow by echocardiography after injection of agitated saline into the right atrium. The later MRI scan could also confirm this initial observation. We could not detect any relevant obstruction of the pulmonary outflow tract. In accordance with about 25% of all L-TGA subjects, our patient presented with meso-/dextrocardia.

The anatomical findings in L-TGA are explained by an incorrect cardiac looping of the truncus arteriosus on the left side around the sinus venosus of the embryonic heart tube, which takes place in the fifth week of embryonic development. Regularly, the separation of the pulmonary trunk and the aorta is the result of a spirally turned septum which develops in the truncus arteriosus. As a consequence of this septal gyration, the later ascending aorta crosses the pulmonary trunk. In L-TGA this rotation is missing so that aorta and pulmonary trunk stay parallel to each other. This anatomic finding is described by the term *side-by-side position*. For the typical congenitally corrected transposition of the great arteries, the aorta stays ventral and left-sided from the pulmonary trunk. Thus, this form of TGA is also called L-, that is, *laevo* TGA. Nevertheless, the exact molecular mechanism of this heart defect still remains unknown.

Given a physiological sequence of pulmonary and systemic blood flow, patients suffering from L-TGA can stay without clinical symptoms for many years. Our patient has never been conspicuous for cardiac reasons before. However, this first episode of acute decompensated heart failure, induced by tachycardic atrial fibrillation, led to the diagnosis of a congenital heart defect at the age of 45 years.

The limiting factor consists in the ongoing strain of the right ventricle [6], which has to carry the burden of the systemic blood pressure. It is found that the degree of the atrioventricular valve insufficiency—here tricuspid valve—correlates with the long-term prognosis in these patients [7]. Finally, most of the L-TGA patients get symptomatic by atrial fibrillation, heart failure, or higher degrees of an atrioventricular block [8].

Acute right heart failure is known to present refractory to medicamentous treatment. Being aware of this fact we can retrospectively understand the prolonged catecholamine dependence to recover from the cardiac shock at admittance. Although the acute trigger, that is, the tachycardic atrial fibrillation, has been eliminated immediately, recovery of the originally weak right ventricle has taken much longer, compared with a regular left ventricle.

In strictly selected cases, a surgical option for the treatment of L-TGA would be a *double-switch* procedure. Here a change of the right and left atrium and a switch of the great arteries will be performed. The aim of this intervention is to reintegrate the left ventricle into the systemic circulation. As in L-TGA, the left ventricle is working for the pulmonary circulation only; it gets hypotrophic. Thus, the optimal point of time for this anatomical correction is located within the first weeks after birth [9]. To train the hypotrophic left ventricle before reintegration into the systemic circulation, a banding of the pulmonary artery can be preceded. The benefit of this surgical treatment—especially for clinically inconspicuous patients presenting with a sufficient right ventricular function—is still discussed controversially.

In our patient, cardiac shock could be treated successfully by rhythm control and heart failure medication. As a relapse of heart failure symptoms is likely, heart transplantation will represent the only curative option in the long run.

## Figures and Tables

**Table 1:** Concomitant anomalies in L-TGA are depicted.

Concomitant anomalies in L-TGA	Incidence (%)
Defect of the ventricular septum [3, 4]	60%–70%
Obstruction of the pulmonary outflow tract [3, 4]	30%–50%
Dysplasia or dislocation of the tricuspid valve [3, 4]	90%
Meso-/dextrocardia [5]	25%
Situs inversus [5]	10%

## Abbreviations

bpm: Beats per minute
ECG: Electrocardiography
g: Gram
h: Hour
l: Litre
L-TGA: Congenitally corrected-transposition of the great arteries
*μ*g: Microgram
mg: Milligram
mL: millilitre
mm: Millimetre
mmHg: Millimetres mercury
MRI: Magnetic resonance imaging
ng: Nanogram
TGA: Transposition of the great arteries.

## References

[1] Bjarke BB, Kidd BS (1976). Proceedings: congenitally corrected transposition of the great arteries: a clinical study of 101 cases. *British Heart Journal*.

[2] Binder T (2002). Echokardiographie aktuell: kongenital korrigierte transposition der großen Gefäße (ccTGA). *Austrian Journal of Cardiology*.

[3] Kirklin JW, Blackstone EH, Tchervenkov CI, Castaneda AR (1992). Clinical outcomes after the arterial switch operation for transposition: patient, support, procedural, and institutional risk factors. *Circulation*.

[4] Paladini D, Volpe P, Marasini M (2006). Diagnosis, characterization and outcome of congenitally corrected transposition of the great arteries in the fetus: a multicenter series of 30 cases. *Ultrasound in Obstetrics and Gynecology*.

[5] Feingold B, O’Sullivan B, Del Nido P, Pollack P (2001). Situs inversus totalis and corrected transposition of the great arteries [I,D,D] in association with a previously unreported vascular ring. *Pediatric Cardiology*.

[6] Dimas AP, Moodie DS, Sterba R, Gill CC (1989). Long-term function of the morphologic right ventricle in adult patients with corrected transposition of the great arteries. *American Heart Journal*.

[7] Beauchesne LM, Warnes CA, Connolly HM, Ammash NM, Tajik AJ, Danielson GK (2002). Outcome of the unoperated adult who presents with congenitally corrected transposition of the great arteries. *Journal of the American College of Cardiology*.

[8] Presbitero P, Somerville J, Rabajoli F, Stone S, Conte MR (1995). Corrected transposition of the great arteries without associated defects in adult patients: clinical profile and follow up. *British Heart Journal*.

[9] Dyer K, Graham TP (2003). Congenitally corrected transposition of the great arteries: current treatment options. *Current Treatment Options in Cardiovascular Medicine*.

